# Interleaved periods of exercise do not enhance visual perceptual learning

**DOI:** 10.1167/jov.25.6.5

**Published:** 2025-05-08

**Authors:** Ken W. S. Tan, Amritha Stalin, Adela S. Y. Park, Kristine Dalton, Benjamin Thompson

**Affiliations:** 1 Centre for Eye and Vision Research Limited, Hong Kong SAR, China; 2 School of Optometry and Vision Science, University of Waterloo, ON, Canada; 3 Liggins Institute, University of Auckland, Auckland, New Zealand

**Keywords:** peripheral crowding, neuroplasticity, perceptual learning, visual training, exercise

## Abstract

Animal models indicate that exercise promotes visual cortex neuroplasticity; however, results from studies that have explored this effect in humans are mixed. A potential explanation for these discrepant results is the relative timing of exercise and the task used to index neuroplasticity. We hypothesized that a close temporal pairing of exercise and training on a vision task would enhance perceptual learning (a measure of neuroplasticity) compared to a non exercise control. Thirty-two participants (mean age = 31 years; range, 20–65; *SD* = 11.1; 50:50 sex ratio) were randomly assigned to Exercise or Non Exercise groups. The Exercise group alternated between moderate cycling along a virtual course and training on a peripheral crowding task (5 minutes each, 1 hour total intervention), and the Non Exercise group alternated between passive viewing of the virtual cycling course and the vision task. The protocol was repeated across 5 consecutive days. Both groups exhibited reduced visual crowding after 5 days of training. However, there was no difference in perceptual learning magnitude or rate between groups. Translation of the animal exercise and visual cortex neuroplasticity results to humans may depend on a range of factors, such as baseline fitness levels and the measures used to quantify neuroplasticity.

## Introduction

Neuroplasticity refers to the brain's ability to reorganize its structure and function (Mateos-Aparicio & Rodríguez-Moreno, 2019). Within the visual cortex, neuroplasticity is pronounced during an early critical or sensitive period when visual input guides visual cortex development (Wiesel & Hubel, 1963). As the brain matures and the critical period closes, visual cortex neuroplasticity declines (Bang, Hamilton-Fletcher, & Chan, 2021; Fronius, Cirina, Ackermann, Kohnen, & Diehl, 2014; Seitz, 2017). However, the visual cortex retains a degree of neuroplasticity in adulthood. This change is evident in real-world scenarios where specialists such as radiologists and sommeliers attain mastery in their fields in adulthood following long periods of training. Their mastery can be attributed to perceptual learning—where improvements in sensory tasks occur through repetitive training that alters neural processing (Gibson, 1963; Gilbert, Sigman, & Crist, 2001; Goldstone, 1998; Jeter, Dosher, Liu, & Lu, 2010; Konorski, 1948). As such, perceptual learning is a convenient model for exploring neuroplasticity in humans.

In recent years, there has been an emergence of evidence indicating that neuroplasticity can be augmented in animal and human adult visual cortex using interventions such as environmental enrichment (Kalogeraki, Pielecka-Fortuna, & Löwel, 2017; Sale et al., 2007), non-invasive brain stimulation (Behrens et al., 2017; Esser et al., 2006; Thompson, Mansouri, Koski, & Hess, 2008), and physical exercise evidenced by both behavioral studies (Lunghi & Sale, 2015) and studies documenting exercise induced changes in neurochemistry (Maddock, Casazza, Fernandez, & Maddock, 2016) that could be conducive for neuroplasticity (Stryker & Löwel, 2018).

Exercise is especially appealing as a neuroplasticity enhancer due to its wide-ranging positive effects on general well-being. For example, exercise has been linked with enhanced cognitive ability (Bugg & Head, 2011; Erickson et al., 2009; Erickson et al., 2010; Erickson et al., 2011) and greater tolerance of stress (Greenwood & Fleshner, 2011; Hoffman, Cohen, Ostfeld, Zohar, & Cohen, 2016). In animal models, exercise promotes a neurochemical environment conducive to neuroplasticity (El-Sayes, Harasym, Turco, Locke, & Nelson, 2019). Aerobic exercise increases the availability of growth factors such as brain-derived neurotrophic factor (BDNF), insulin-like growth factor (IGF-1), and vascular endothelial growth factor (VEGF) (Cotman, Berchtold, & Christie, 2007). Together, these neurochemicals induce gliogenesis (formation of new glial cells), neurogenesis (formation of new neurons), synaptogenesis (formation of new synapses), and angiogenesis (formation of new blood vessels) (El-Sayes et al., 2019).

Unlike animal models that tend to consistently show enhanced neuroplasticity following exercise, findings in humans have been mixed (Abuleil, Thompson, & Dalton, 2022). While some studies support the notion that exercise can facilitate neuroplasticity (Holzschneider, Wolbers, Röder, & Hötting, 2012; Lunghi & Sale, 2015; Perini, Bortoletto, Capogrosso, Fertonani, & Miniussi, 2016; Virathone, Nguyen, Dobson, Carter, & McKendrick, 2021), others do not (Campana, Fongoni, Astle, & McGraw, 2020; Connell, Thompson, Green, Sullivan, & Gant, 2018; Finn, Baldwin, Reynaud, & Hess, 2019; Zhou, Reynaud, & Hess, 2017).

One possible explanation for the variability in the results from previous studies is the timing between the exercise and the neuroplasticity measure. Although the optimal sequencing and dosing of exercise for visual perceptual learning (VPL) enhancement are unknown, previous studies investigating VPL have predominantly employed acute exercise paradigms (Campana et al., 2020; Connell et al., 2018; Perini et al., 2016). We hypothesized that alternating short periods of exercise with short periods of training on a visual task would enable the neurochemical changes produced by exercise to be retained during each training epoch and enhance the magnitude and rate of VPL. An alternating approach was chosen because simultaneous exercise and vision task training was technically challenging due to the requirements for a stable head position, stable fixation, and use of eye-tracking.

Participants were trained on a crowding task conducted in peripheral vision, similar to that employed by Hussain, Webb, Astle, and McGraw (2012), who demonstrated a robust reduction in crowding following training. We hypothesized that alternating exercise (cycling on a stationary bike) with this perceptual learning task would lead to greater reductions in crowding at the trained location than a control group who experienced alternating periods of rest and perceptual learning. As is the case with many VPL tasks that show specificity for trained visual field location (Lu & Dosher, 2022), we expected that learning at an untrained location would be similar between the Exercise and Non Exercise groups.

We chose a crowding perceptual learning task because gamma-aminobutyric acid (GABA)-mediated lateral inhibitory connections may contribute to visual crowding (Raveendran, Tsang, Tiwana, Chow, & Thompson, 2020), and exercise has been shown to alter the visual cortex excitation–inhibition balance in animal models (Baroncelli et al., 2012; Kaneko & Stryker, 2014). Additionally, we were interested in potential future clinical applications of exercise-enhanced perceptual learning and crowding, given that crowding contributes to vision deficits in individuals with macular degeneration. Because sex differences in neuroplasticity mechanisms may exist (Barha & Liu-Ambrose, 2018; Chaieb, Antal, & Paulus, 2008; Kuo, Paulus, & Nitsche, 2006), we also assessed whether sex at birth influenced the effect of exercise on perceptual learning.

## Methods

### Participants

Thirty-two naïve observers participated in the experiment and were recruited across two sites, the Centre for Eye and Vision Research in Hong Kong (HK) and the School of Optometry and Vision Science at the University of Waterloo, Canada (CA) (*n* = 16 at each site; 16 males and 16 females across both sites). All participants had normal or corrected-to-normal visual acuity, defined as a visual acuity (VA) of <0.1 logMAR with a difference of no more than 0.1 logMAR between the eyes, and they were in good general health. Participation was voluntary, and written consent was obtained prior to commencement of experiments. The study was approved by the ethics boards of both the University of Waterloo and The Hong Kong Polytechnic University and was in accordance with the tenets of the Declaration of Helsinki. Participants were compensated for attending study sessions.

### Apparatus

At the HK site, stimuli were generated in Python and presented in PsychoPy Version 2021.2.3 (Peirce, 2007; Peirce, 2009) on an Intel Core i7-12700K, 3.6 GHz, 12-core CPU (Intel Corporation, Santa Clara, CA, USA) with 32 GB of RAM and drawn on the frame buffer (24 GB) of a GeForce RTX 3090 GPU (NVIDIA, Santa Clara, CA, USA). Visual stimuli were presented on an Asus ROG Swift OLED PG42UQ monitor (screen resolution, 3840 × 2160, 61.69° × 37.14°; refresh rate, 120 Hz; ASUSTeK Computer, Taipei, Taiwan) at a viewing distance of 77 cm. The viewing distance was maintained with a head and chin rest, and at this distance each pixel subtended 0.96′ of visual angle. Display luminance was measured with a Konica Minolta LS-150 luminance meter (Konica Minolta, Tokyo, Japan) and linearized using the inverse Gamma function.

At the CA site, stimuli were also presented in PsychoPy Version 2021.2.3 on an Intel Core i3-4160 CPU (8 GB RAM) and drawn on an Intel HD Graphics 4400. Visual stimuli were presented on a V241p monitor (screen resolution, 1920 × 1080, 42.51° × 24.68°; refresh rate, 60 Hz; HP, Palo Alto, CA, USA) at a viewing distance of 67 cm (stimulus visual angles were scaled to be identical across both sites). Viewing distance was also maintained with a head and chin rest and at this distance each pixel subtended 1.33′ of visual angle. Display luminance was measured with a Konica Minolta CS-100 luminance and color meter and linearized using the inverse Gamma function. At both sites, participant responses were recorded with the corresponding letter keys on a keyboard by the researcher.

At the HK site, fixation stability was monitored binocularly (500 Hz) with an EyeLink Portable Duo infrared eye tracker (SR Research, Kanata, ON, Canada), spatial resolution accuracy of 0.01°. At the CA site, fixation stability was also detected binocularly (90 Hz) using a Tobii Eye Tracker 4C (Tobii AB, Stockholm, Sweden), spatial resolution accuracy of ∼0.5°.

At both sites the visual scene used while cycling was presented on an LG 55-inch OLED55C1PCB television (LG Corporation, Seoul, South Korea) and was generated with the Zwift cycling app (Zwift, Long Beach, CA, USA). At the HK site, the Tacx NEO Bike Smart Trainer (Garmin, Olathe, KS) was used for cycling, and at the CA site the Renpho RQ002 (Renpho, Irvine, CA, USA) was used. Bikes were placed 135 cm in front of the television screen and were linked with the Zwift app such that the visual scene moved in accordance with pedaling, and the tactile feel of the observed road was relayed to the bike. Studies of exercise-induced visual cortex neuroplasticity in animal models have previously observed that concurrent visual input during exercise results in greater neuroplasticity enhancements than exercise without concurrent visual input (Kaneko & Stryker, 2014). Participants in the Non Exercise group were presented with a video of a similar visual scene during their rest blocks, played and viewed from the same distance. At both sites, prior to visual training, each participant's height and weight were taken with a measuring tape and weighing scale. All participants completed the Queen's College Step Test using a plyometric box 16.25 inches in height. Heart rate (HR) measures were obtained with a Garmin Vivosmart 4/Venu Sq fitness tracker.

### Stimuli

Visual stimuli used for this study were 10 Sloan optotypes: C, D, H, K, N, O, R, S, V, and Z. Sloan letters are an optotype typically used to test visual acuity and have been designated as the US standard for acuity testing by the National Academy of Sciences, National Research Council, Committee on Vision (National Research Council, 1980). The optotype scales easily, as it has a 1:1 aspect ratio and each stroke width is 1/5 its side. Letters used were black (HK: 3.53 cd/m^2^; CA: 0.71 cd/m^2^) and presented on a gray background of mean luminance (HK: 88.16 cd/m^2^; CA: 56.3 cd/m^2^). The fixation target was a white cross (0.2°) presented in the center of the screen.

### Tasks

#### Queen's college step test

The Queen's College Step Test (McArdle, Katch, Pechar, Jacobson, & Ruck, 1972) is a submaximal aerobic fitness test used to estimate maximum oxygen uptake (VO_2_max). While wearing the HR monitor, participants stepped on and off a plyometric box in an “up–up–down–down” manner for 3 minutes. An audible metronome was played in the background corresponding to either 96 beats per minute (bpm) for males or 88 bpm for females. Participants were asked to move each foot in sync with the audible beat. The HR reading of the individual was taken 20 seconds after completion of the test (i.e., at the 3-minute, 20-second mark) and was used to benchmark a loaded HR for the Exercise group. It was also used to calculate the loaded HR as a proportion of maximum HR.

The age-predicted maximal heart rate (Fox, Naughton, & Haskell, 1971) was used to calculate the maximum HR (see Equation 1):

(1) $$\begin{array}{c} {\mathrm{HR}}_{Max}=220-Age\; \end{array}$$

The proportion of the step-test HR relative to the maximum HR is specified in Equation 2:

(2) $$\begin{array}{c} \%\mathrm{HR}=\frac{\mathrm{HR}}{HR_{Max}}\; \end{array}$$

#### Letter size

The letter size used for the main crowding task was determined separately for each participant (as described below). Randomly selected, single (uncrowded) Sloan letters centered 4° above central fixation were shown for 150 ms. While maintaining central fixation, participants verbally identified the presented letter. Utilizing the descending and ascending method of limits, the researcher changed the letter size in steps of 0.025° to determine an estimate of the participant's threshold for identifying the uncrowded letter. The researcher was able to verify the participant's response with the answer shown on the bottom right of the screen out of sight of the participant. When an estimated threshold had been determined, the letter size was then increased by one step above threshold, and the participant was asked to correctly identify five letters at this size. In the event of errors, the letter size was further increased by one step and the process repeated. Upon correct identification of five consecutive letters, the letter size was doubled. Participants were then asked to correctly identify 10 consecutive letters at the doubled size. If no errors were reported, this size was then used as a starting letter size for the peripheral crowding task. As before, if an error was made, the letter size was increased by one step and the process was repeated. This method ensured that the size of the letters used during the peripheral crowding task were well above discrimination threshold and any incorrect responses were not due to an inability to resolve the letter. This task was completed binocularly.

#### Peripheral crowding task

**Figure 1.**
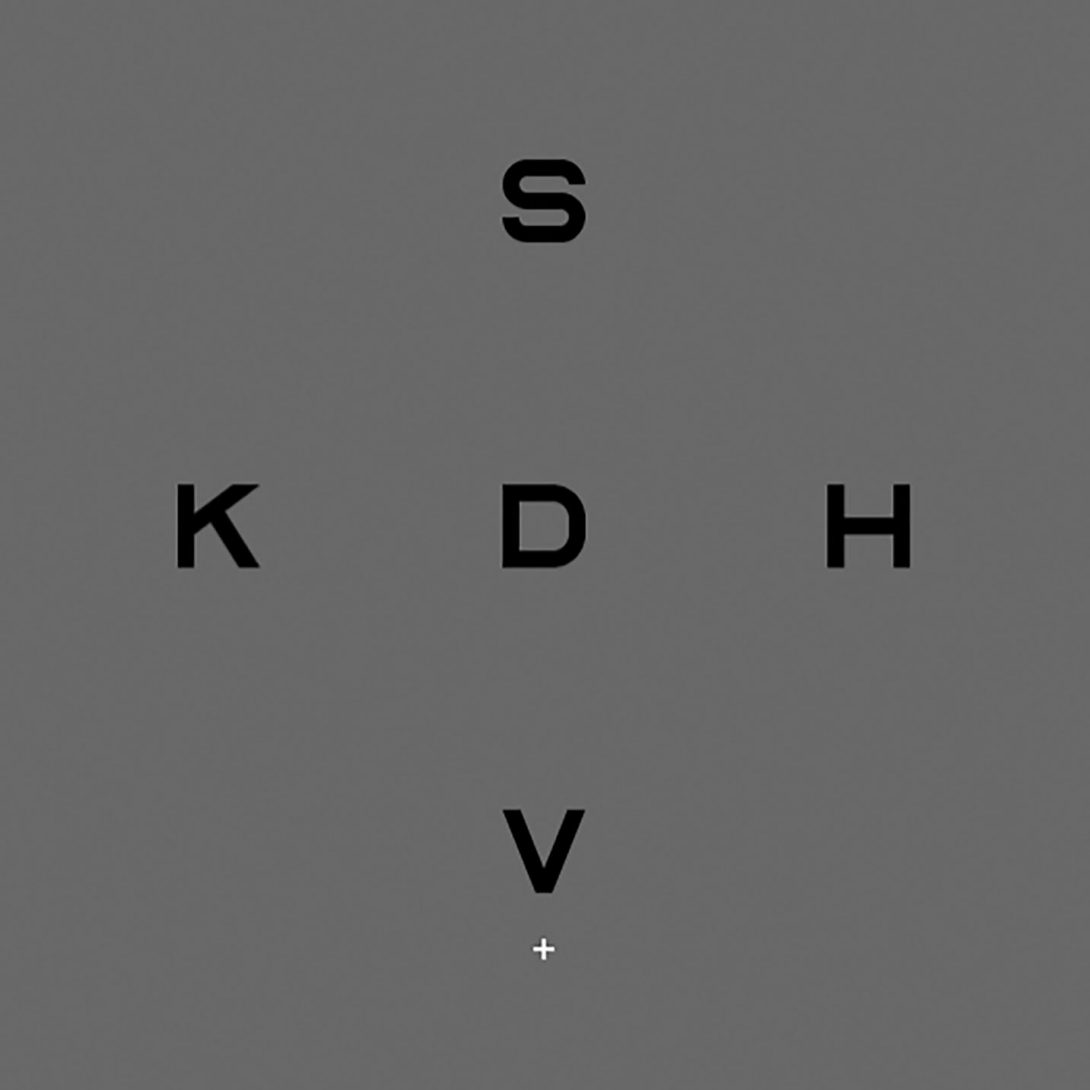
Example of stimuli used in the peripheral crowding task (top condition). Five Sloan letters were randomly selected without replacement and presented in a cross formation. The target letter was positioned in the center of the formation centered 4° vertically from fixation. Depending on the condition (top or bottom) this could be either above or below fixation. Participants were asked to report the target letter while maintaining central fixation. Letter separation between flankers and target (measured center to center) was equal and varied in response to a correct/incorrect identification of the target.

A peripheral crowding task similar to that utilized by Hussain et al. (2012) was employed to determine peripheral crowding performance. The task involved presentation of five Sloan letters (from the set of 10). The target letter appeared in the center and was flanked in each cardinal direction by one of the four other letters, resulting in a cross formation (see Figure 1). On each trial, letters were randomly sampled without replacement to ensure no letters were repeated.

Distances (measured center to center) between flankers and the target letter were equal and yoked. There were two conditions: top and bottom. Depending on the condition, letters appeared above or below the central fixation. The center of the target letter was always vertically displaced, 4° from fixation. Only the top condition was used for VPL.

Letter distance was controlled by a 3-down 1-up staircase (79.4% percentile threshold). Correct responses reduced the letter distance, but incorrect responses increased it in step sizes of 0.2°. The staircase consisted of 90 trials, and the mean of the last six reversals was used to determine threshold. The minimum and maximum letter separations were fixed at “letter size” and “4°,” respectively. If performance improved to the point that the threshold for letter distance matched letter size for two consecutive training sets, then letter size was reduced by 10% for the following set.

The task involved participants fixating binocularly on a fixation cross that appeared for 500 ms followed by the stimuli appearing for 150 ms. Participants had 3000 ms to verbally report the target central letter and were told to respond as quickly as possible. On hearing the response, the researcher would press the corresponding letter key on a backlit keyboard (in the darkened room). The researcher pressing letter keys was shielded from the display to prevent bias. If no response was entered in the 3000 ms allotted, then the trial automatically timed out with an incorrect response. Auditory feedback was given for all responses (correct/incorrect). Each run consisted of 90 trials and took approximately 5 minutes. Crowding performance was quantified by taking the ratio of the letter separation to the letter size, where a smaller ratio indicated better performance.

### Procedure

The protocol was run over 5 consecutive days. On day 1, prior to experiment commencement, participants gave written consent for participation. Following this, they were asked to wear a HR monitor. The logMAR visual acuity was then measured and assessed to ensure that the inclusion criterion was met. Anthropometric measurements (height and weight) were also taken. Next, participants completed a general health questionnaire and two pre-physical activity screenings: the International Physical Activity Questionnaire–Short Form (IPAQ-SF) (Craig et al., 2003) and the Get Active Questionnaire (Canadian Society for Exercise Physiology, 2017) to determine their individual level of physical activity participation, general health, and fitness. Each participant's HR was measured at rest, 15 minutes after donning the HR monitor.

Following this, participants completed the letter size determination task followed by baseline measurements of performance on the peripheral crowding task for the top and bottom conditions. After baseline measures had been taken, participants completed the Queen's College Step Test and their active HR was measured; participants in the Exercise group had to maintain this HR during the exercise component. Participants who were unable to successfully complete the step test were excluded. This was to ensure a base level of fitness. Eligible participants were sequentially assigned to a group (Exercise or Non Exercise) from a randomized list (generated prior to the recruitment of the first participant) upon successful completion of the step test. The training protocol commenced following a 15-minute rest period.

**Figure 2.**
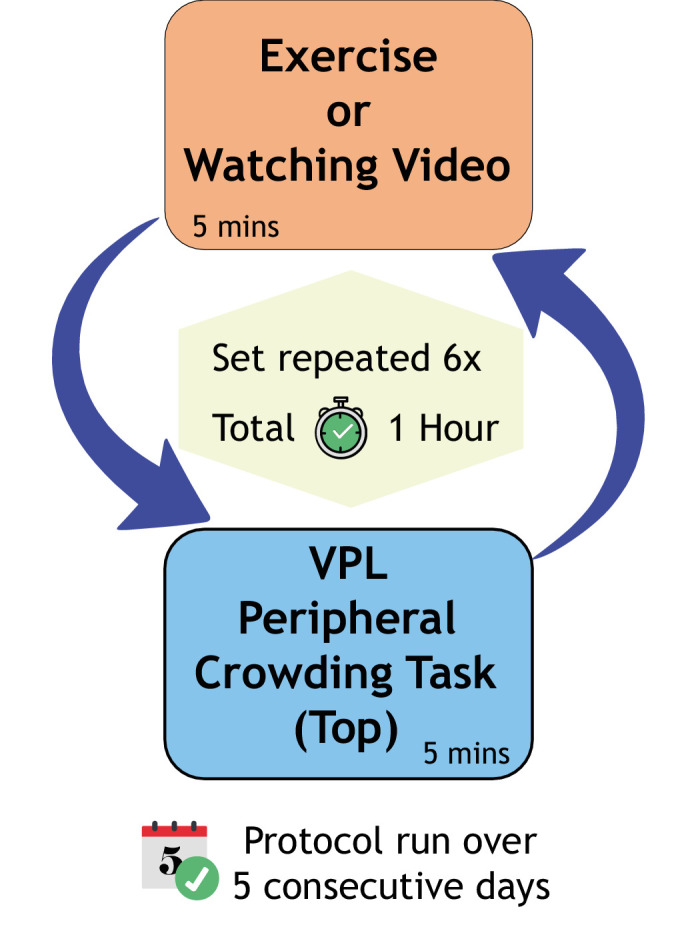
Sequence of events for the training protocol. Participants either cycled at their step test HR or watched a video for 5 minutes followed by the peripheral crowding task for 5 minutes. This was repeated for 1 hour. Only the top condition was used for the VPL.

Participants followed the training protocol sequence shown in Figure 2. Participants in the Exercise group were asked to attain (as quickly as possible) and maintain their active HR. Participants in the Non Exercise group watched a video of a visual scene similar to that viewed by the Exercise group. Eye movements were monitored with an eye tracker during the peripheral crowding task to ensure that participants were not foveating the target letter. Eye movements within 1.5° from central fixation were considered valid fixations. This threshold was chosen to allow for any movement the participant might make while responding verbally. The purpose of fixation monitoring was to ensure that the peripheral crowding task was being completed as intended and that the participants were not foveating the target letter. Participants were excluded if their daily fixation rate was <70% across the six daily sessions. Short breaks could be taken as required, after the perceptual learning task and before the cycling or video watching. On the last day, after the final session, participants were given a 15 minute break before post-measures were taken for both top and bottom conditions.

## Results

This study involved 32 participants across two sites (mean age = 31.38 ± 11.07; mean ± SD), with a 50:50 split of males and females. The Exercise group had nine males and seven females, and the Non Exercise group had nine females and seven males. Data were combined across both sites for analyses. The mean %HR for the step test of the Exercise and Non Exercise groups were 0.62 ± 0.14 and 0.68 ± 0.11 (mean ± SD), respectively. The mean starting letter sizes of the Exercise and Non Exercise groups were 0.94 ± 0.02 and 0.92 ± 0.02 (mean ± SD), respectively.

Analyses were performed using R Version 4.3.1 (R Core Team, 2023). A visual inspection of *Q*-*Q* plots indicated that some portions of the dataset were not normally distributed—baseline measures: top condition ratios (*W* = 0.95, *p* = 0.13) and bottom condition ratios (*W* = 0.75, *p* < 0.001); post-measures: top condition ratios (*W* = 0.93, *p* = 0.04) and bottom condition ratios (*W* = 0.78, *p* < 0.001). Transformation did not help to normalize the dataset so non-parametric analyses were used, supplemented by Bayes factors where possible (calculated using JASP) (JASP, 2024).

**Figure 3.**
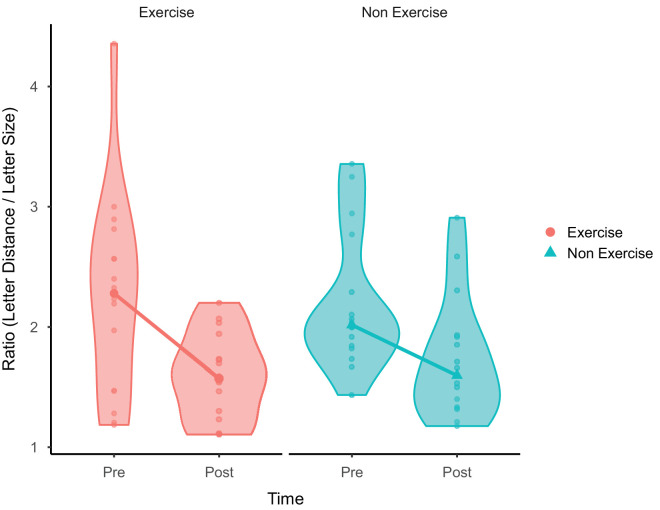
Top condition medians with violin plots. Both groups showed an effect of perceptual learning; however, there was no statistical difference between the groups.

Two-sample paired Wilcoxon tests were run to compare performance between baseline measures and post-measures of both groups in the trained location (top). Both the Exercise group (*W* = 66, *p* < 0.001, effect size *r* = 0.98 [*BF*_10_ = 313.77, *W* = 134, *R-hat* = 1.10], median = 2.28 [pre-] and 1.57 [post], interquartile range [IQR] = 1.47–2.63 [pre-] and 1.29–1.79 [post]) and the Non Exercise group (*W* = 54, *p* = 0.003, effect size *r* = 0.73 [*BF*_10_ = 22.72, *W* = 122, *R-hat* = 1.05], median = 2.02 [pre-] and 1.60 [post], IQR = 1.84–2.41 [pre-] and 1.33–1.92 [post]) showed a difference between baseline and post measures indicating an effect of PL. However, a Wilcoxon rank-sum test showed no statistical difference between the pre- and post differences of the two groups (*W* = 112, *p* = 0.56, effect size *r* = 0.10 [*BF*_10_ = 0.45, *W* = 112, *R-hat* = 1.00]), indicating no effect of alternating exercise and visual training. See Figure 3 for a visual representation.

**Figure 4.**
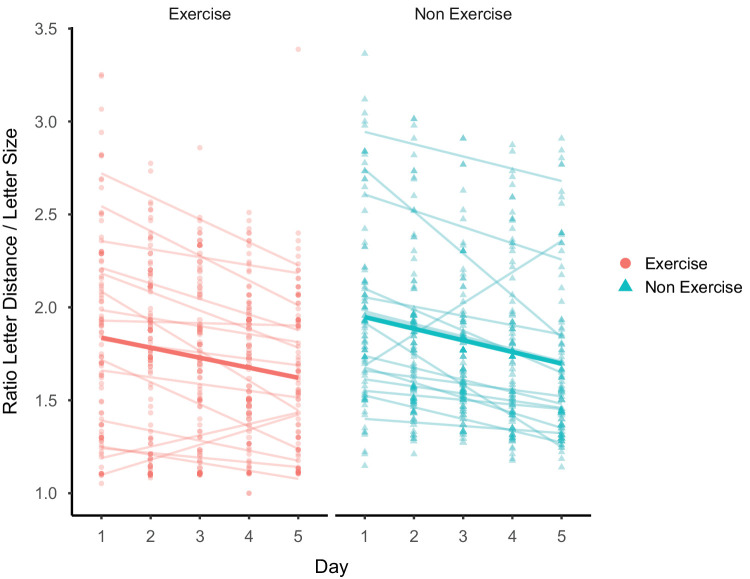
Learning slopes for each group. Individual data and slopes are shown in more transparent colors. The bold line represents the group slope. The rates of learning were not statistically different between the groups.

Linear regression models were fitted to each participant's daily sessions to obtain slopes determining the rates of learning (see Figure 4). Slope values representing task performance across daily sessions were calculated using the daily mean. A comparison between the groups did not show a statistical difference between the rates of learning (mean slope and SD values for the Exercise and Non Exercise groups were −0.09 ± 0.14 and −0.14 ± 0.08, respectively; *t* = −0.31, *p* = 0.76).

**Figure 5.**
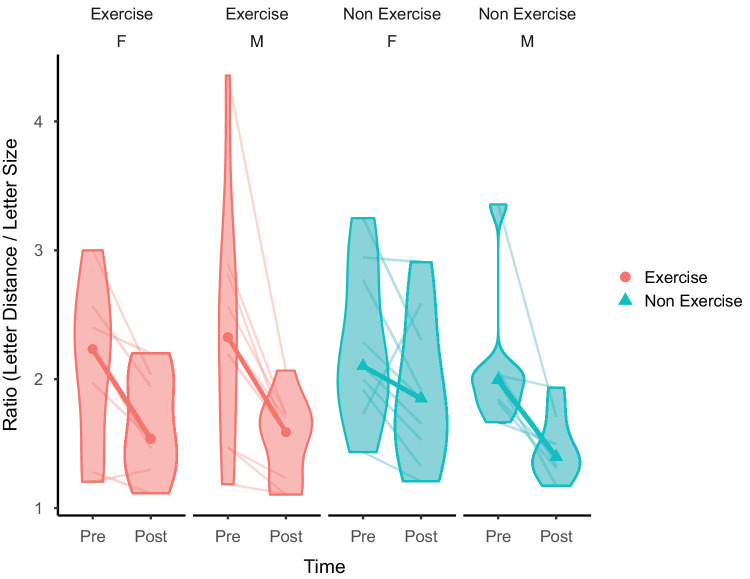
Medians and violin plots for both groups, split by sex. Bold lines indicate the group medians, and lower transparency lines indicate individual data.

To determine if there was an effect of biological sex, the dataset was split by sex, and two-sample paired Wilcoxon tests were run for each group (see Figure 5). There was no difference in the amount of task improvement between males and females for either the Exercise group (*W* = 20, *p* = 0.25, effect size *r* = 0.29 [*BF*_10_ = 0.74, *W* = 43.00, *R-hat* = 1.00], median= −0.63 [male] and −0.43 [female], IQR = −1.17 to −1.37 [male] and −0.70 to −0.18 [female]) or the Non Exercise group (*W* = 26, *p* = 0.61, effect size *r* = 0.13 [*BF*_10_ = 0.51, *W* = 37.00, *R-hat* = 1.00], median = −0.51 [male] and −0.44 [female], IQR = −0.78 to −0.31 [male] and −0.59 to −0.22 [female]).

**Figure 6.**
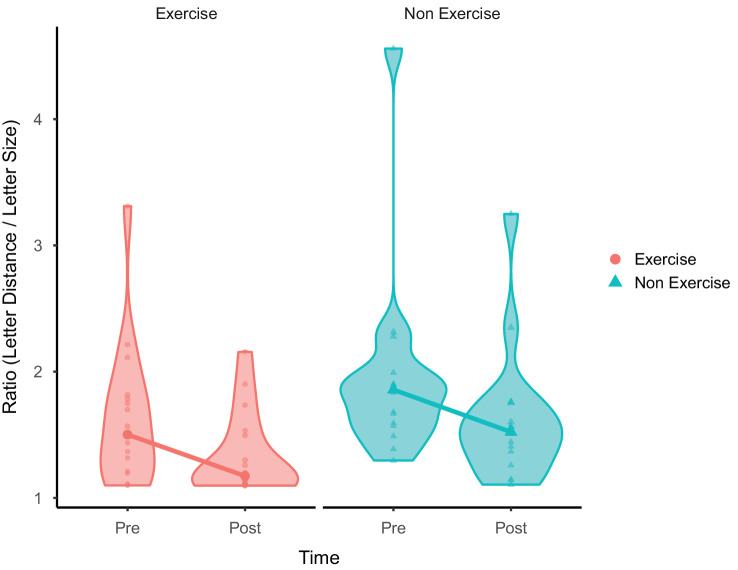
Bottom condition medians with violin plots. Both groups demonstrated transfer of perceptual learning; however, there was no statistical difference between the groups.

To determine if the extent of VPL transfer was affected by exercise, performance for the bottom condition was analyzed (see Figure 6). Both the Exercise group (*W* = 28, *p* = 0.007, effect size *r* = 0.67 [*BF*_10_ = 33.027, *W* = 96, *R-hat* 1.03], median = 1.50 [pre-] and 1.17 [post], IQR = 1.21 to 1.79 [pre-] and 1.11 to 1.50 [post]) and Non Exercise group (*W* = 62, *p* = 0.001, effect size *r* = 0.80 [*BF*_10_ = 122.476, *W* = 130, *R-hat* = 1.02], median = 1.86 [pre-] and 1.52 [post], IQR = 1.59 to 1.92 [pre-] and 1.34 to 1.75 [post]) showed a difference between baseline and post measures indicating a transfer of VPL. However, a Wilcoxon rank-sum test indicated that there was no statistical difference in improvement between the two groups (*W* = 148, *p* = 0.462, effect size *r* = 0.13 [*BF*_10_ = 0.38, *W* = 148, *R-hat* = 1.00], median = −0.17 [Exercise] and −0.29 [Non Exercise], IQR = −0.34 to −0.06 [Exercise] and −0.47 to −0.14 [Non Exercise]).

To determine whether the amount of VPL differed across location, the pre- and post differences in VPL for the top (trained location) and bottom (untrained condition) were compared with Wilcoxon rank-sum tests. A larger VPL (median difference = −0.30) was observed for the Exercise group compared with the Non Exercise group (median difference = −0.12). For the Exercise group, *W* = −55.0, *p* = 0.003, effect size *r* = 0.75 [*BF*_10_ = 31.47, *W* = 13.0, *R-hat* = 1.03], median = −0.61 (top) and −0.17 (bottom), IQR = −0.87 to −0.23 (top) and −0.34 to −0.06 (bottom). For the Non Exercise group, two zero-difference pairs were removed from analyses and the Wilcoxon signed-rank test was completed with 14 pairs, such that *W* = −17.5, *p* = 0.003, effect size *r* = 0.28 [*BF*_10_ = 0.48, *W* = 35.0, *R-hat* = 1.00], median = −0.46 (top) and −0.29 (bottom), IQR = −0.76 to −0.18 (top) and −0.36 to −0.13 (bottom). However, these differences between the Exercise and Non Exercise groups (16 pairs) were not statistically significant (*W* = 81.0, *p* = 0.08, effect size *r* = 0.44 [*BF*_10_ = 0.766, *W* = 81.0, *R-hat* = 1.00], median = −0.30 [Exercise] and −0.05 [Non Exercise], IQR = −0.60 to −0.15 [Exercise] and −0.27 to −0.06 [Non Exercise].

## Discussion

The effect that exercise exerts on visual cortex neuroplasticity has been investigated by various research teams; however, findings have been mixed. One of the earlier studies (Lunghi & Sale, 2015) reported that exercise boosted the shift in eye dominance induced by short-term monocular deprivation, an index of homeostatic plasticity within the visual system. However, this effect has not been replicated consistently since. Although some studies have reported an enhancing effect of exercise on measures of visual cortex neuroplasticity (Perini et al., 2016; Virathone et al., 2021), others have not (Campana et al., 2020; Chen, Hall, Bobier, Thompson, & Chakraborty, 2022; Connell et al., 2018). One commonality of these studies, though, is that the exercise component employed was delivered in a block. The current study differed from previous work that tested whether alternating exercise and vision training would have an effect on neuroplasticity. The results indicated no effect of exercise. Biological sex also did not influence the effect of alternating exercise on VPL. A VLP duration of 5 consecutive days was chosen because robust VPL effects can occur within this timeframe (Hawkey, Amitay, & Moore, 2004; Larcombe, Kennard, & Bridge, 2017), and fewer consecutive sessions are more suitable for potential clinical translation. However, it is possible that exercise effects may have emerged within a longer term training protocol.

All VPL training was completed in the top location; however, improvements were observed from pre- to post in the bottom location, indicating task transfer to an untrained location. This was interesting given that specificity, or failure to transfer, is a hallmark of VPL (Ahissar & Hochstein, 1996; Fiorentini & Berardi, 1980; Shiu & Pashler, 1992), and performance enhancements attained during PL tasks often fail to generalize to alternative, related tasks or stimuli (Jeter, Dosher, Petrov, & Lu, 2009). Webb, Roach, and McGraw (2007) demonstrated that transfer can be enhanced if tasks retain commonalities in judgment characteristics (separate from simple visual attributes, such as retinal position or orientation). Indeed, in the current study, the judgment of the crowded letter was similar for the top and bottom, albeit in a different retinal location, which might explain the transfer effects observed. Another plausible explanation of transfer comes from the work by Xiao et al., (2008) , which demonstrated that transfer can be achieved by “double training.” For the current study, double training could have been instigated by the baseline measurement of the untrained bottom location which then became “primed” for transfer.

When comparing across locations for each group, we observed that there appeared to be enhanced learning for the trained location in the Exercise compared to the Non Exercise group; however, the group difference was not statistically significant.

Despite not finding an effect of alternating exercise, we observed that performance on a peripheral crowding task could be enhanced after a period of VPL, replicating the findings reported by Hussain et al. (2012). Crowding represents a bottleneck of object perception (Levi, 2008) and is usually investigated at peripheral locations because it is more pronounced in peripheral vision (Bouma, 1970). Peripheral crowding was employed in the current study, as this study is part of a longer term project investigating vision rehabilitation. In unimpaired vision states, crowding is a phenomenon that typically occurs in peripheral vision. However, in impaired vision states, it further exacerbates the already reduced visual function for conditions such as amblyopia (Levi & Klein, 1985) and age-related macular degeneration (Wallace, Chung, & Tjan, 2017). Evidence that crowding occurs even when target and flankers are presented to separate eyes (Flom, Heath, & Takahashi, 1963) suggests that the phenomenon occurs beyond retinal processing and involves a combination of information within the cortex. This is congruent with observations that performance for a crowding task can be improved with VPL (suggesting a neural change) and demonstrates the potential for VPL to be used as a tool to rehabilitate vision for deficits that result from peripheral crowding. The observation of transfer to an untrained location also suggests that VPL of a crowing task may generalize to other visual field locations in a rehabilitation context.

## Conclusions

Visual perceptual learning reduced crowding, but this effect was not enhanced by exercise, suggesting that interleaved exercise did not enhance neuroplasticity. However, there was transfer of learning observed from trained to untrained locations, highlighting the potential for perceptual learning to rehabilitate deficits resulting from peripheral crowding.
